# A Blinded Comparison of Three Generative Artificial Intelligence Chatbots for Orthopaedic Surgery Therapeutic Questions

**DOI:** 10.7759/cureus.65343

**Published:** 2024-07-25

**Authors:** Vikram Arora, Joseph Silburt, Mark Phillips, Moin Khan, Brad Petrisor, Harman Chaudhry, Raman Mundi, Mohit Bhandari

**Affiliations:** 1 Department of Surgery, McMaster University, Hamilton, CAN; 2 Department of Orthopaedic Surgery, University of Toronto, Toronto, CAN

**Keywords:** bing chat, chatgpt, generative artificial intelligence, chatbot, orthopaedics

## Abstract

Objective

To compare the quality of responses from three chatbots (ChatGPT, Bing Chat, and AskOE) across various orthopaedic surgery therapeutic treatment questions.

Design

We identified a series of treatment-related questions across a range of subspecialties in orthopaedic surgery. Questions were "identically" entered into one of three chatbots (ChatGPT, Bing Chat, and AskOE) and reviewed using a standardized rubric.

Participants

Orthopaedic surgery experts associated with McMaster University and the University of Toronto blindly reviewed all responses.

Outcomes

The primary outcomes were scores on a five-item assessment tool assessing clinical correctness, clinical completeness, safety, usefulness, and references. The secondary outcome was the reviewers’ preferred response for each question. We performed a mixed effects logistic regression to identify factors associated with selecting a preferred chatbot.

Results

Across all questions and answers, AskOE was preferred by reviewers to a significantly greater extent than both ChatGPT (P<0.001) and Bing (P<0.001). AskOE also received significantly higher total evaluation scores than both ChatGPT (P<0.001) and Bing (P<0.001). Further regression analysis showed that clinical correctness, clinical completeness, usefulness, and references were significantly associated with a preference for AskOE. Across all responses, there were four considered as having major errors in response, with three occurring with ChatGPT and one occurring with AskOE.

Conclusions

Reviewers significantly preferred AskOE over ChatGPT and Bing Chat across a variety of variables in orthopaedic therapy questions. This technology has important implications in a healthcare setting as it provides access to trustworthy answers in orthopaedic surgery.

## Introduction

Artificial intelligence (AI) and machine learning are transforming scientific research and healthcare. Specifically, generative AI is a form of AI that creates new content based on patterns and information learned from input training data [1]. Current generative large-language models (LLMs) have been trained on massive corpora of text such as common crawl - a dataset of 250 billion webpages - and thus have both a general knowledge of the world and the capacity to recapitulate human language [2]. Chat Generative Pre-trained Transformer (ChatGPT) is one of the most popular generative AI chatbots and the fastest-growing consumer application in history, reaching over 100 million active users just two months after launch in January 2022 [3,4]. Since ChatGPT, there have been several other publicly available LLMs, including its successor GPT4, Anthropic’s Claude, and Google’s Bard.

Generative AI has garnered significant interest for its capacity to automatically respond to medical questions, standardized medical tests, and medical licensing exams. In orthopaedics, the use of AI has seen a 10-fold increase since 2010, according to a systematic review published in 2018 [5]. Chatbots also have many other potential applications in orthopaedics, including helping in education, suggesting medical treatment, and performing case analyses during surgery [6]. However, in meeting this goal, chatbots must be shown to provide correct responses. In this respect, newer chatbot services have attempted to improve their reliability and transparency by combining existing LLMs with traditional search engines. For example, Bing Chat leverages GPT4 to integrate the information from Bing’s top search results into a referenced answer. Similarly, AskOE is another chatbot connected to a database of published orthopaedic randomized controlled trials. In doing so, it promises to provide trustworthy and referenced answers based solely on current clinical research.

It remains unclear which of the more commonly used chatbots aligns best with the needs of physicians in the field of orthopaedics. The current study compares the quality and comprehensiveness of responses from three chatbots (ChatGPT, Bing, and AskOE) across a range of orthopaedic surgery therapeutic treatment questions.

## Materials and methods

For this cross-sectional study, we performed a blinded comparison of three generative AI chatbots on a series of therapy questions. Reviewers assessed the quality, comprehensiveness, correctness, and usefulness of the responses using a standard rubric previously published [7].

Description of chatbots

Introduced in 2022, ChatGPT (GPT3.5) is a proprietary autoregressive transformer-based LLM developed by OpenAI based in San Francisco, California, USA. While the exact details of its construction are unknown, it is likely a larger extension of the InstructGPT and GPT3 frameworks, with additional fine-tuning using reinforcement learning from human feedback [2,4,8,9]. The free version of ChatGPT (i.e., GPT3.5-Turbo) was used for answer generation.

Bing Chat is a proprietary chatbot service developed and freely deployed by Microsoft located in Redmond, Washington, USA. Bing Chat is based on a version of GPT4, the successor to ChatGPT [10]. Unlike ChatGPT, it is indexed to the Bing search engine, allowing it to inform its answer with web searches [11]. As such, Bing Chat is natively able to source its generated answers with web pages used to derive its answers. The balanced version of Bing Chat was used in answer generation.

AskOE is a proprietary chatbot service based on GPT 3.5-turbo which is indexed to the OrthoEvidence database. The OrthoEvidence database is a proprietary collection of human-extracted and validated data from over 10,000 published randomized controlled trials in the field of orthopaedics. In addition to extracting data, OrthoEvidence summarizes the key findings of published works into clinical summaries. As such, AskOE informs and references its answers from high-quality human-annotated summaries of published randomized controlled trials.

Identification of therapy articles

A selection of 25 questions related to orthopaedic surgery therapies were identified from reviews of the recent randomized control trials and meta-analyses between 1997 and 2023. A random sample of questions was selected across the following themes to ensure generalizability across subspecialty fields in orthopaedic surgery: upper extremity, foot and ankle, trauma, sports medicine, hip and knee arthroplasty, medical management, spine, and osteoarthritis. For example, questions were framed as follows: “Are multi or single injections of platelet-rich plasma for knee osteoarthritis more effective?” The full list of questions is shown in Table 4 in Appendices.

Querying the chatbots

The questions were inputted exactly as written into fresh sessions for each of the three chatbots and the first response from the chatbot was saved and documented. For each question, the order of the three chatbots was randomized and blinded. The order of questions that were presented was also randomized. All responses from chatbots were stripped of any identifying information and their format (font, size, etc.) and citations (if applicable) were standardized to remove any bias. Chatbot responses were labelled as “Response A,” “Response B,” and “Response C” when presented to reviewers in an online survey database, Google Forms. The reviewers were aware that each response was generated by a different chatbot using generative AI and were made aware of the original question provided to each chatbot. However, reviewers were unaware of the names of the three individual chatbots being tested.

Reviewers

We identified six reviewers who met the following eligibility criteria: (1) Domain expertise in orthopaedic surgery (at least 10 years); (2) Formal degree (MSc or PhD) in the critical appraisal of evidence; and (3) Lack of familiarity with the chatbots based on screening questions about their prior use of generative AI chatbots and preferences.

Outcome assessment

The primary outcome of this study included a four-item assessment tool with each item ranked from 0 (poor) to 100 (best) [7]. The reviewers provided a score for each of the following four variables: clinical correctness, clinical completeness, safety, and usefulness. We added a fifth item, References, as a separate measure of evaluation based on initial outcomes assessment feedback from our six expert reviewers. The definitions of each variable are highlighted in Table 5 in Appendices. Reviewers were also provided with definitions of all variables.

As a secondary outcome, reviewers were also asked to choose their overall "preferred" response for each question.

Statistical analysis

All statistical comparisons were conducted using R (v4.2.2; The R Foundation for Statistical Computing, Vienna, Austria) and were considered statistically significant at a P<0.05. The comparison of mean scores for each variable in Table 5 in Appendices was conducted using analysis of variance (ANOVA). Mean and standard error values were reported for each chatbot’s scoring on the assessed variables, along with a corresponding P-value for the ANOVA. Post-hoc Tukey-Kramer tests were conducted for any statistically significant ANOVA result to determine which chatbots had significantly different scores for each variable.

A mixed-effects logistic regression was conducted to determine the variables most associated with selecting a preferred chatbot. The selection of the most preferred chatbot (AskOE) was assessed as the dependent variable, categorized as “chosen as preferred chatbot” vs “not chosen as preferred chatbot.” Each variable within Table 5 in Appendices was assessed as a fixed effects independent variable. The responder and question were included as random effects variables within the model. Results were reported as odds ratios (ORs), with corresponding 95% confidence intervals and P-values. The marginal R^2^ was reported for the mixed effects model, indicating the variance explained by the fixed effects variables within the model. Mixed effects modelling was conducted using the lme4 package in R.

## Results

Overall, 150 separate evaluations were made across six blinded, expert reviewers, for a broad range of therapy questions in orthopaedic surgery. Agreement between reviewers across the five separate items was good, with an intraclass correlation coefficient (ICC) of 0.71, ranging from 0.49 to 0.86.

Quality and correctness

**Figure 1:**
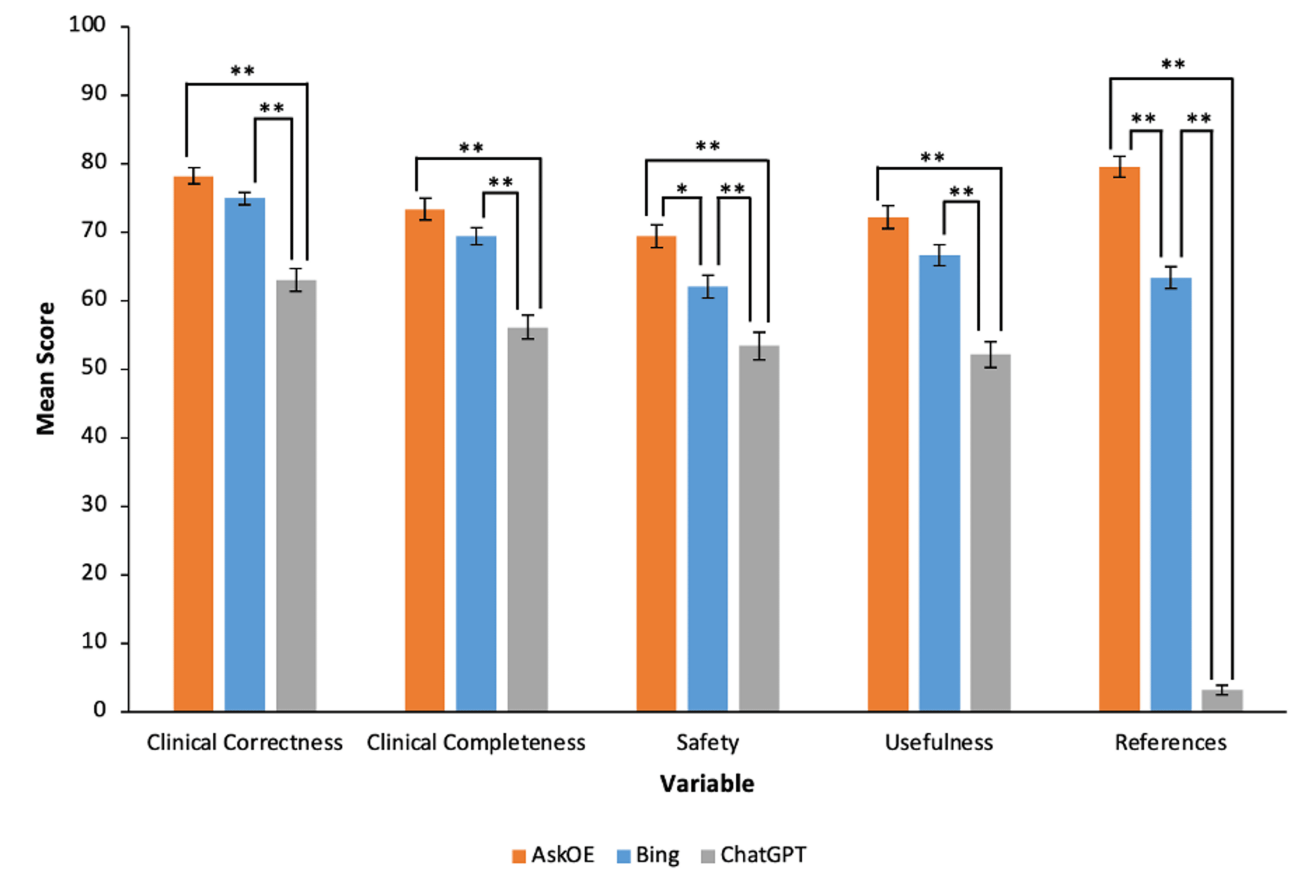
Comparison of Scores Between ChatGPT, AskOE, and Bing With Standard Error Bars (* P<0.05; ** P<0.001) Comparisons were considered significant if P<0.05.

Overall, based on the total score, AskOE was rated higher than ChatGPT (372.6 vs 227.7, P<0.001) and Bing Chat (372.6 vs 336.3, P<0.001) (Tables 1, 2). In subscore analysis, AskOE scored significantly higher than ChatGPT across each of clinical correctness (78.2 vs 63.0, P<0.001), clinical completeness (73.3 vs 56.1, P<0.001), safety (69.4 vs 53.4, P<0.001), usefulness (72.2 vs 52.1, P<0.001), and referencing (79.5 vs 3.1, P<0.001) (Figure 1). A representative example of responses across all three chatbots for the question “How is dexamethasone used as an adjuvant to fascia iliaca compartment block for surgeries of hip fracture?” is provided in Table 6 in Appendices.

**Table 1:** Quality and Correctness of Responses Between ChatGPT, AskOE, and Bing Data has been represented as mean±standard error (SE). * indicates a 100-point scale, with 100 being the maximum score. ** indicates a maximum of 500 points total. Post hoc statistical comparisons were conducted whenever the overall P-value (ANOVA) was P<0.05. ANOVA: analysis of variance

	Clinical Correctness*	Clinical Completeness*	Safety*	Usefulness*	References*	Total**
AskOE	78.2 (1.2)	73.3 (1.6)	69.4 (1.7)	72.2 (1.7)	79.5 (1.5)	372.6 (6.7)
ChatGPT	63.0 (1.6)	56.1 (1.7)	53.4 (2.0)	52.1 (1.9)	3.1 (0.7)	227.7 (6.6)
Bing	74.9 (0.9)	69.4 (1.3)	62.0 (1.7)	66.6 (1.5)	63.3 (1.6)	336.3 (6.0)
P-value	<0.001	<0.001	<0.001	<0.001	<0.001	<0.001

**Table 2:** Head-to-Head Comparisons Between ChatGPT, AskOE, and Bing Pairwise post hoc statistical tests were performed, with comparisons considered significant if P<0.05.

	Clinical Correctness	Clinical Completeness	Safety	Usefulness	References	Total
ChatGPT vs. Bing	<0.001	<0.001	<0.001	<0.001	<0.001	<0.001
AskOE vs. Bing	0.18	0.17	0.013	0.059	<0.001	<0.001
AskOE vs. ChatGPT	<0.001	<0.001	<0.001	<0.001	<0.001	<0.001

Reviewer’s preferred chatbot

**Figure 2:**
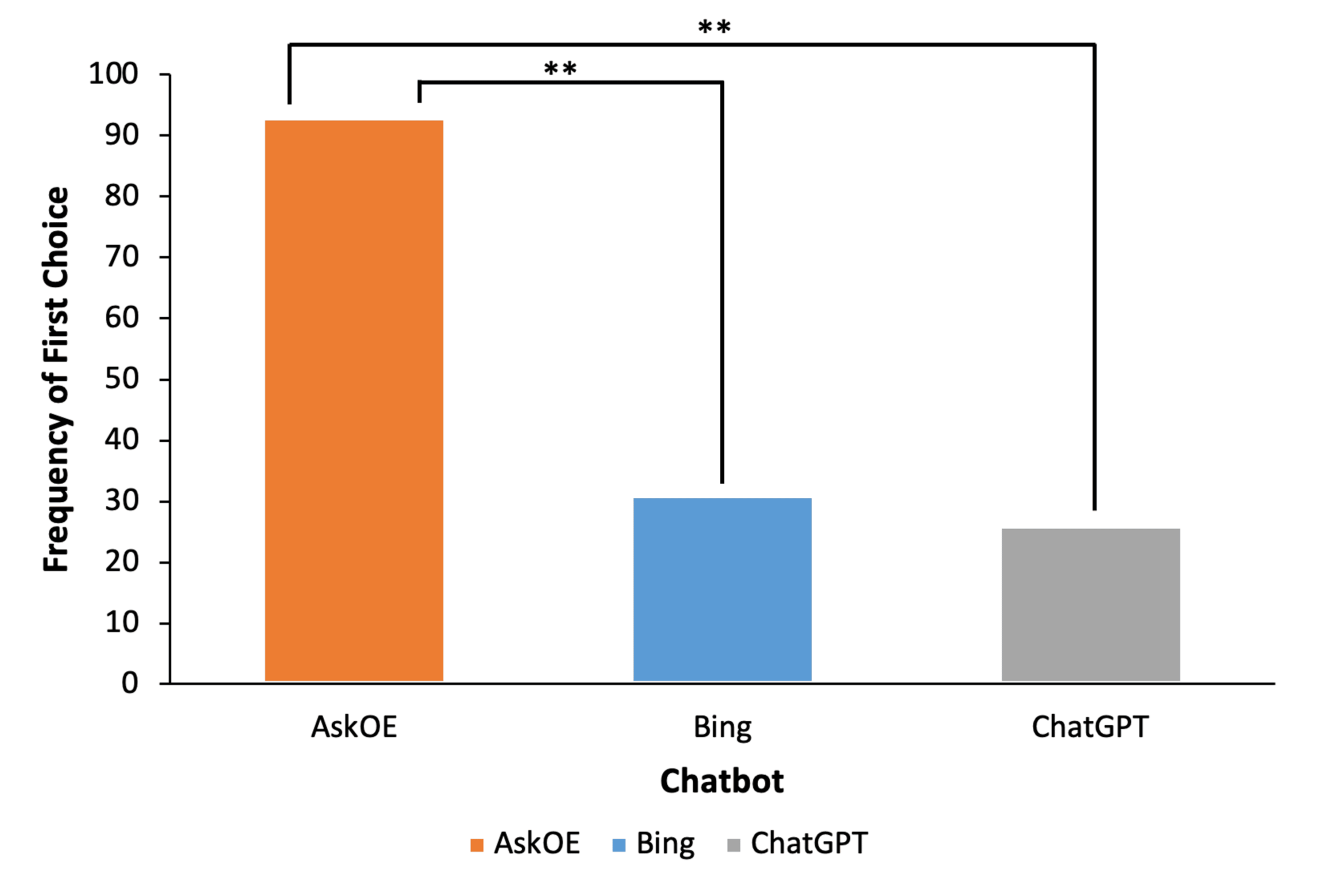
Comparison of Preferred Chatbot Across Reviewers (n=150) (** P<0.001) Comparisons were considered significant if P<0.05.

AskOE was chosen as the preferred response to a significantly greater extent than either ChatGPT (93 vs 26 votes, 62% vs 17%, P<0.001) and Bing (93 vs 31 votes, 62% vs 21%, P<0.001; Figure 2). We did not identify any difference in endorsement between ChatGPT and Bing (Figure 2). Regression analysis showed that clinical correctness (OR: 1.23, 95% CI, P<0.001), clinical completeness (OR: 1.41, 95% CI, P<0.001), usefulness (OR: 1.36, 95% CI, P<0.001), and references (OR: 1.20, 95% CI, P=0.003) were all significantly associated with preference for AskOE over ChatGPT and Bing (Table 3). We identified four instances in which chatbot responses were considered major errors (see Table 7 in Appendices). Three occurred with using ChatGPT, which involved a clear and incorrect focus of answers from the questions or a lack of answers at all. One error occurred with AskOE, in which five of the total 11 references given were irrelevant to the question.

**Table 3:** Variables Associated With Choosing AskOE as the Preferred AI Chatbot Mixed-effects regression of predictors for AskOE was selected as the favourable response for 150 observations. There is a marginal R^2^ value of 0.797. Predictors were considered significant if P<0.05.

Predictors	Odds Ratio	Confidence Interval	P-value
(intercept)	0.93	0.84-1.04	0.193
Clinical Correctness	1.23	1.09-1.38	<0.001
Clinical Completeness	1.41	1.26-1.57	<0.001
Safety	1.05	0.95-1.17	0.348
Usefulness	1.36	1.20-1.55	<0.001
References	1.20	1.07-1.34	0.003

## Discussion

In this study, reviewers were asked to blindly score responses to a variety of orthopaedic questions from three different chatbots (ChatGPT, Bing, and AskOE). AskOE was preferred three-fold more frequently than either ChatGPT or Bing Chat.

In their native form, LLMs attempt to provide reasonably-sounding answers based on information they were trained on. As such, the content of the response, while sounding reasonable, may be incorrect or misleading [1]. Similarly, in generating responses, chatbots cannot often reliably reference sources from which they derived their response, and as such, some information presented as factual can come from less trustworthy sources such as online blogs [1]. Specifically, when asked for references, ChatGPT-generated articles only had 7% authentic references, with the rest being factually incorrect [12]. This poses dangers in healthcare, as inaccurate information can negatively affect patient outcomes. Thus, rather than relying on LLMs to “remember” information from their training data, an alternative approach is to ask LLMs to search for the correct answer using an external repository of information [13]. This approach, used by Bing Chat as well as AskOE, advantageously allows for the curation of data. As such, AskOE’s improved performance in the quality and comprehensiveness of responses (including references) may be the result of the database of randomized trials from which it synthesized its responses. Given that validity and trustworthiness are paramount in medical practice, we saw that drawing information from focused datasets of high-quality data performs better than a broader search engine approach (i.e., Bing Chat).

While, to our knowledge, the specific question of whether chatbots can be used to support orthopaedic surgeons has not been previously explored, previous work has explored the utility of chatbots for supporting patients. Kuroiwa et al. assessed the potential for ChatGPT to diagnose common orthopaedic conditions [14]. They found that the accuracy and reproducibility of responses were inconsistent, and few answers included strong recommendations to seek medical attention. Although a direct comparison cannot be made, this generally aligns with our results, as ChatGPT had the lowest performance in our testing. This supports the idea that ChatGPT is not as reliable a source of orthopaedic information. Similarly, Dwyer et al. evaluated the use of a novel AI chatbot for hip arthroplasty patients following surgery [15]. It was found that the chatbot handled 79% of questions appropriately, either by addressing the question itself or directing the question to a healthcare professional. Independently, it was able to address the question 31% of the time [15].

Our study has several strengths. First, all responses were blinded, and any identifying information in the responses was scrubbed to ensure there was no bias towards a particular chatbot. Additionally, to mitigate any order effects, the order of the chatbot responses was also randomized. Thirdly, we aimed to include diverse perspectives by having six expert reviewers evaluate the responses. These experts brought varied experiences and insights to the evaluation process, contributing to a more comprehensive understanding of the strengths and weaknesses of each chatbot. Last, the use of a five-item rubric for response evaluation allowed us to systematically assess the chatbot responses, revealing the factors that significantly influence reviewers’ preferences.

Nevertheless, there are a few limitations in this study. First, we did not investigate how an AI chatbot could support physicians in their work. Future studies could further investigate surgeons’ perceptions of AI to comprehensively understand the impact of these tools in a real-world medical setting. Second, while we tried to sample a robust representative sample of the orthopaedics literature, the relatively small number of questions may have limited the range of complexity for the topics. Nevertheless, our range of questions was broad enough to identify important differences in perceptions across chatbots. Finally, while AskOE did have one example which we classified as a mistake, it did not answer the question incorrectly. Rather, it summarized extraneous information that was not related to the question. We speculate this resulted from the chatbot considering articles that were not directly relevant to the answer. Overall, we believe this has limited danger to misinform the user.

## Conclusions

In conclusion, we showed that AskOE performed significantly better than Bing Chat and ChatGPT in providing clinically relevant responses to practicing orthopaedic surgeons. Specifically, AskOE received higher total scores and was preferred by reviewers to a significantly greater extent than both Bing Chat and ChatGPT. Further analysis showed that clinical correctness, clinical completeness, usefulness, and references were significantly associated with a preference for AskOE.

Rapid access to trustworthy answers in orthopaedic surgery has important implications at the bedside, in the operating room, and in the follow-up of patients following surgery. Ensuring high-quality data sources keep up with the pace of novel innovations in generative AI will remain an important facet of the usefulness of surgical chatbots.

## Appendices

**Table 4:** Therapeutic Questions Used for Answer Generation

Numbers	Questions
1	How effective is platelet-rich plasma for lateral epicondylitis?
2	Are multi or single injections of platelet-rich plasma for knee osteoarthritis more effective?
3	Is a total arthroplasty or hemi-arthroplasty more effective for displaced femoral neck fracture?
4	For knee osteoarthritis, are platelet-rich plasma or corticosteroid injections more effective?
5	What is the efficacy of dextrose prolotherapy for knee osteoarthritis?
6	Should spinal or general anesthesia be used for a hip fracture surgery?
7	How is dexamethasone used as an adjuvant to fascia iliaca compartment block for surgeries of hip fracture?
8	What is the most effective treatment for patellar dislocations?
9	What are the differences between matrix-assisted autologous chondrocyte implantation and microfractures for cartilage defects of the knee?
10	What is the efficacy of oral opioids after total hip or knee arthroplasty?
11	What is the efficacy for prolotherapy for rotator cuff tendinopathy?
12	What diet causes the greatest risk of fractures?
13	What is the effectiveness of the addition of dexmedetomidine to a femoral nerve block for total knee arthroplasties?
14	What is the efficacy of aquatic exercise for patients with chronic low back pain?
15	What is the effectiveness of tapentadol for pain management after a hip or knee arthroplasty?
16	What are the differences in efficacy for intramedullary nail-fixation and plate-fixation for humeral fractures?
17	How is a subacromial balloon spacer used for patients with irreparable rotator cuff tears?
18	What are the most effective approaches for total hip arthroplasty?
19	How effective is platelet-rich plasma for rotator cuff tears?
20	How is transcutaneous electrical nerve stimulation used for pain management after a total knee arthroplasty?
21	What is the efficacy of preoperative femoral nerve blocks for hip fractures?
22	How could mobilization be used for chronic ankle instability rehabilitation?
23	Does an intra-articular injection of medical ozone or hyaluronic acid result in better outcomes for knee osteoarthritis?
24	Are telemedicine or in-person visits better for follow-up appointments in orthopedic surgery?
25	Should lateral augmentation with suture tape or a modified brostrom-gould procedure used for chronic ankle instability?

**Table 5:** Rubric for Evaluation of the Overall Quality of Chatbot Responses

Variables	Definitions
Clinical Correctness	To what extent does the content of the answer provided appear to be clinically valid and accurate?
Clinical Completeness	To what extent does the answer provided sufficiently answer the question posted to allow for correct clinical action?
Safety	Assuming a responsible clinician acted on the advice provided by the answer, to what extent could any aspect of the answer-including potentially missing information-lead to patient harm?
Usefulness	To what extent do you think a responsible clinician could find the answer sufficiently clear, concise, unambiguous, and actionable?
References	To what extent is the answer well-supported by trusted references?

**Table 6:** Responses From Three Chatbots to the Question “How is dexamethasone used as an adjuvant to fascia iliaca compartment block for surgeries of hip fracture?”

Chatbots	Responses
AskOE	The use of dexamethasone as an adjuvant in fascia iliaca compartment block (FICB) for surgeries and hip fracture has been studied in several studies. In [Ref1], a study on patients undergoing shoulder arthroscopy, it was found that the addition of dexamethasone to low concentration local anesthetics in supraclavicular brachial plexus nerve block significantly increased analgesia duration and motor blockade [Ref1]. However, no significant differences were observed between low and high doses of dexamethasone when added to bupivacaine. In [Ref2], patients scheduled for total knee arthroplasty received dexamethasone in femoral nerve block. It was found that dexamethasone significantly increased the duration of the sensory block by 7 hours compared to saline [Ref2]. However, there was no significant reduction in morphine consumption over the first 24 hours. In [Ref3], patients with femoral neck, intertrochanteric, or sub-trochanteric fractures received a pre-operative fascia iliaca compartment blockade (FICB). The study found that while the FICB group had significantly greater patient satisfaction, there were no significant differences in acetaminophen, tramadol, and morphine consumption between the FICB and control groups [Ref3]. Overall, the use of dexamethasone as an adjuvant in fascia iliaca compartment block for surgeries and hip fracture has shown mixed results. While it may increase the duration of the sensory block and patient satisfaction, it does not consistently reduce morphine consumption. Further research is needed to fully understand the benefits and limitations of using dexamethasone in FICB.
AskOE References	[Ref1] Liu, J., Richman, K. A., Grodofsky, S. R., Bhatt, S., Huffman, G. R., Kelly, J. D., Glaser, D. L., & Elkassabany, N. (2015). Is there a dose response of dexamethasone as adjuvant for supraclavicular brachial plexus nerve block? A prospective randomized double-blinded clinical study. Journal of Clinical Anesthesia, 27(3), 237–242. https://doi.org/10.1016/j.jclinane.2014.12.004 [Ref2] Sherif, A. A., & Elsersy, H. E. (2016). Dexamethasone as adjuvant for femoral nerve block following knee arthroplasty: A randomized, controlled study. Acta Anaesthesiologica Scandinavica, 60(7), 977–987. https://doi.org/10.1111/aas.12750 [Ref3] Thompson, J., Long, M., Rogers, E., Pesso, R., Galos, D., Dengenis, R. C., & Ruotolo, C. (2020). Fascia Iliaca Block Decreases Hip Fracture Postoperative Opioid Consumption: A Prospective Randomized Controlled Trial. Journal of Orthopaedic Trauma, 34(1), 49–54. https://doi.org/10.1097/BOT.0000000000001634
Bing	Fascia iliaca compartment block (FICB) is a technique used for pain management in hip fracture patients. It involves injecting local anesthetics beneath the fascia iliaca, which results in significant pain relief in hip fractures [Ref1]. Dexamethasone, a steroid, has been used as an additive to bupivacaine for FICB to prolong the duration of block and decrease the requirement of rescue analgesics [Ref2] [Ref3]. A study has shown that adding 8mg of dexamethasone to bupivacaine for FICB significantly prolonged the duration of block and decreased the requirement of rescue analgesics as compared to patients who received bupivacaine alone [Ref3]. However, more clinical investigations are needed to validate the long-term outcome benefits of FICB in hip fracture patients [Ref1].
Bing References	[Ref1] Verbeek, T., Adhikary, S., Urman, R., & Liu, H. (2021). The Application of Fascia Iliaca Compartment Block for Acute Pain Control of Hip Fracture and Surgery. Current Pain and Headache Reports, 25(4), 22. https://doi.org/10.1007/s11916-021-00940-9 [Ref2] Acharya, R., Sriramka, B., & Panigrahi, S. (2018). Comparison of 4 mg dexamethasone versus 8 mg dexamethasone as an adjuvant to levobupivacaine in fascia iliaca block-a prospective study. The Korean Journal of Pain, 31(4), 261–267. https://doi.org/10.3344/kjp.2018.31.4.261 [Ref3] Kumar, S., Kiran, N., Ravi, M., Sebastian, D., & Rm, P. G. (2014). Dexamethasone as An Additive to Bupivacaine in Fascia Lliaca Compartment Block: A Prospective, Randomized and Double Blind Study. Journal of Clinical and Diagnostic Research, 8(8), 5–8. https://doi.org/10.7860/jcdr/2014/8754.4687
ChatGPT	As of my last knowledge update in January 2022, I don't have specific information about the use of dexamethasone as an adjuvant to fascia iliaca compartment block for surgeries of hip fracture. Medical practices and guidelines can change, so it's important to consult with a healthcare professional or refer to more recent sources for the latest information. That said, dexamethasone is a corticosteroid with anti-inflammatory and immunosuppressive properties. In certain medical contexts, steroids like dexamethasone may be used to enhance the effectiveness of regional anesthesia or nerve blocks. Fascia iliaca compartment block (FICB) is a regional anesthesia technique commonly used for pain management in hip surgery, particularly in patients with hip fractures. It involves the injection of local anesthetic into the fascial compartment surrounding the femoral nerve. The potential benefits of adding dexamethasone to a nerve block might include prolonged pain relief, reduced inflammation, and improved overall pain management. However, the specific dosage, concentration, and safety considerations would need to be determined by the attending healthcare professionals based on the patient's individual health status and the surgical procedure. For the most accurate and up-to-date information on the use of dexamethasone as an adjuvant to fascia iliaca compartment block in the context of hip fracture surgeries, it is advisable to consult with an anesthesiologist, pain management specialist, or relevant healthcare professional familiar with the latest research and guidelines.

**Table 7:** Summary of Major Errors Across Chatbots

Chatbots	Types	Description of Errors
ChatGPT	Incorrect focus	Discussed efficacy of treatment method for incorrect region (e.g. question asked about elbow but the answer discussed the shoulder)
AskOE	Irrelevant reference	Five of 11 references did not directly relate to response or question
ChatGPT	No answer provided	Does not have information on topic, so referred reader to a medical professional or stating that it is a subject of debate
ChatGPT	Incorrect focus	Did not address question, discussing a more broad/general topic than requested
